# Efficacy of endovascular therapy for stroke in developing country: A single-centre retrospective observational study in Indonesia from 2017 to 2021

**DOI:** 10.1016/j.heliyon.2023.e23228

**Published:** 2023-12-04

**Authors:** Mohammad Kurniawan, Kevin Mulya Saputri, Taufik Mesiano, Reyhan E. Yunus, Affan P. Permana, Septo Sulistio, Eka Ginanjar, Rakhmad Hidayat, Al Rasyid, Salim Harris

**Affiliations:** a Department of Neurology, Dr. Cipto Mangunkusumo Hospital, Faculty of Medicine, Universitas Indonesia, Jakarta, Indonesia; b Department of Radiology, Dr. Cipto Mangunkusumo Hospital, Faculty of Medicine, Universitas Indonesia, Jakarta, Indonesia; c Department of Neurosurgery, Dr. Cipto Mangunkusumo Hospital, Faculty of Medicine, Universitas Indonesia, Jakarta, Indonesia; d Department of Emergency Medicine, Dr. Cipto Mangunkusumo Hospital, Faculty of Medicine, Universitas Indonesia, Jakarta, Indonesia; e Department of Internal Medicine, Dr. Cipto Mangunkusumo Hospital, Faculty of Medicine, Universitas Indonesia, Jakarta, Indonesia

**Keywords:** Endovascular therapy, Mechanical thrombectomy, Ischemic stroke, Indonesia, Developing countries

## Abstract

**Background:**

Indonesia as a developing nation faces a plethora of challenges in applying endovascular therapy (EVT), mostly due to the lack of physicians specialized in neuro-intervention, high operational cost, and time limitation. The efficacy of EVT in improving functional outcomes of stroke in developing countries has not been previously studied.

**Methods:**

This retrospective cohort study was conducted at Dr. Cipto Mangunkusumo Hospital (Jakarta, Indonesia) from January 2017 to December 2021. Large vessel occlusion (LVO) diagnosis was established based on a combination of clinical and imaging characteristics. We assessed patients’ functional independence on day-90 based on modified Rankin Scale (mRS) between the endovascular treatment group and the conservative group (those receiving intravascular thrombolysis or medical treatment only). Functional independence was defined as mRS ≤2.

**Results:**

Among 111 stroke patients with LVO, we included 32 patients in the EVT group and 50 patients in the conservative group for this study. Patients with younger age (p = 0.004), lower hypertension rate (p < 0.001), higher intubation rate (p = 0.014), and earlier onset of stroke were observed in the EVT group. The proportion of mRS ≤2 at day-90 in the EVT group was higher than the conservative group (28.1 % vs. 18.0 %; p = 0.280). Patients within mRS ≤2 group had earlier onset-to-puncture time (p = 0.198), onset-to-recanalization time (p = 0.341), lower NIHSS (p = 0.026) and higher ASPECTS (p = 0.001) on admission. In multivariate analysis, ASPECTS (aOR 2.43; 95%CI 1.26–4.70; p = 0.008) defined functional independence in the EVT group.

**Conclusion:**

The endovascular therapy group had a higher proportion of mRS ≤2 at day-90 than the conservative group despite its statistical insignificance.

## 1 Introduction

Indonesia recorded a more than one-and-a-half-fold increase in stroke prevalence within five years and spent over 150 million USD for its treatment in 2018 [1,2]. Acute ischemic stroke (AIS) accounted for two-thirds of stroke cases in Indonesia, including at our institution, Dr. Cipto Mangunkusumo Hospital (CMH) [3,4]. Furthermore, large vessel occlusion (LVO) constituted one-third of AIS resulted in major disability and death [5,6]. Recanalization therapy in LVO has been the gold standard of treatment as long as the criteria are met [7].

Endovascular therapy (EVT) application in daily practice has significant disparity compared to its guideline in research setting. Trained medical staff, high cost, poor referral system, and limited hospital facilities are the major challenges faced in developing countries, such as Indonesia [8]. The cost of EVT devices in Indonesia is nearly 40 % higher due to import tax. This condition further limits the number of EVT providers across the nation [9]. Intra-hospital time delay also contributed in EVT outcome, since the chance of complete recanalization was reduced by 13 % hourly from door-to-puncture time [10].

Our institution is a tertiary teaching hospital located in Central Jakarta. “Code Stroke” was introduced to our hospital with an aim to improve hyperacute stroke service and provide a better outcome. The historical first intravenous thrombolysis (IVT) was performed in 2014. Later on, EVT was introduced in 2017 with a more structured system.

To our knowledge, there has been no study from developing countries regarding the comparison of EVT versus conservative therapy in improving good functional outcomes in AIS patients. In this study, we aimed to investigate EVT efficacy in a resource-limited setting. Our hypothesis was LVO patients treated with EVT still had greater functional independence than those in the conservative group.

## 2 Methods

This retrospective open-label observational study was conducted at CMH, Jakarta, Indonesia. We reviewed all AIS patients treated in our institution from January 2017 to December 2021.

All patients who presented with stroke to the emergency department were assessed for their demographic characteristics, medical history, vital signs, neurological examinations, NIH stroke severity (NIHSS), laboratory tests, electrocardiogram, and 64-slice brain CT scan without contrast.

The inclusion criteria in this study were adults aged over 18-years-old without hyperdense lesions found on their first brain CT scan, NIHSS between 6 and 25, and pre-stroke mRS less than 1. Patients were excluded from our analysis if they had incomplete medical record data, occlusion in the posterior circulation, and poor systemic condition (renal and hepatic failure, systemic malignancy, or burn injury). All non-contrast brain CT scan, including Alberta Stroke Program Early CT (ASPECT) score, was retrospectively reviewed by a neuroradiologist who was blinded to the patients’ outcome.

The ‘Code stroke’ team consisted of consultant physicians and residents (specialty trainee doctors) of Neurology, Neurosurgery, Radiology, and Emergency Departments; nurses; radiographers; and pharmacists. Multimodal imaging methods, including CT angiogram and CT perfusion, were not available. We were blinded to the baseline collateral and penumbra data. Emergency team sent patient's data and brain CT to the responsible consultant. The diagnosis of LVO was established based on a combination of patient's clinical presentations and imaging results. Routine radiological identification was early ischemic changes (EIC) in stroke patients.

Patients in the EVT group received recanalization therapy with EVT, with or without intravenous thrombolysis (IVT). Those in the conservative group received IVT or medical treatment only. The final decision for patient's treatment plan was determined at the discretion of fellow consultant neurologists and a neurosurgeon. Each patient was individually assessed whether they were suitable for EVT or not. The primary time window for EVT was within 8 h since the onset of symptoms. In case of clinical and CT-scan mismatch, this window period could be extended up to 24 h. The primary reasons for conservative treatment in AIS were profund EIC, late arrival beyond time window, treatment refusal by the patient, and the presence of comorbidities.

A written informed consent was obtained from a spouse or first-degree family member before procedure. When there were no exclusion criteria identified, intravenous tPA (Alteplase) was administered within 6 h of onset. The Alteplase dose we administered was 0.6 mg/kgBW of which 10 % dose was given as intravenous (IV) bolus followed by continuous IV infusion within 1 h. The Neuro-intervention medical team in our hospital consists of two consultant neurologists (MK, RH) and a neurosurgeon (AP). Arteriotomy access was performed on the right femoral artery. Stent retriever (Solitaire, Irvine, CA, USA) was the only option to recanalize occlusion. When it was unavailable, modified techniques and tools were determined by operator's discretion. Successful recanalization rate was equivalent to thrombolysis in cerebral infarction (TICI) scores 2b/3. Patients in the EVT group were then admitted to the Intensive Care Unit (ICU) after the procedure and underwent CT examination in 24 h. All patients in this study received standard medical therapy according to the AHA/ASA guidelines for AIS. The Trial of ORG 10172 in Acute *Stroke* Treatment (TOAST) criteria was used to determine stroke etiology (large artery atherosclerosis-LAA, cardiac emboli, or undetermined).

### 2.1 Outcome

We followed up the patients through telephone interviews either with the patients themselves, their spouse or other family member to assess mRS at day-90 post procedure. Good functional outcome was defined as mRS ≤2. This study had been granted approval from the institutional ethical board of Faculty of Medicine, *Universitas Indonesia* number 21-09-0917. Supporting data presented in this study are available upon reasonable request to the corresponding author.

### 2.2 Statistical analysis

The data were analyzed using SPSS version 20.0 (IBM, New York). For categorical data, either χ2-tests or Fisher exact tests were used when appropriate. Student-t-test or Mann-Whitney-U test was used for continuous data. Statistical significance was considered when 2-sided *P-*value was <0.05. Two most significant factors with p < 0.20 were further tested by multivariate logistic regression analysis to determine factors associated with functional independence after 90 days post-treatment.

## 3 Results

**Fig. 1:**
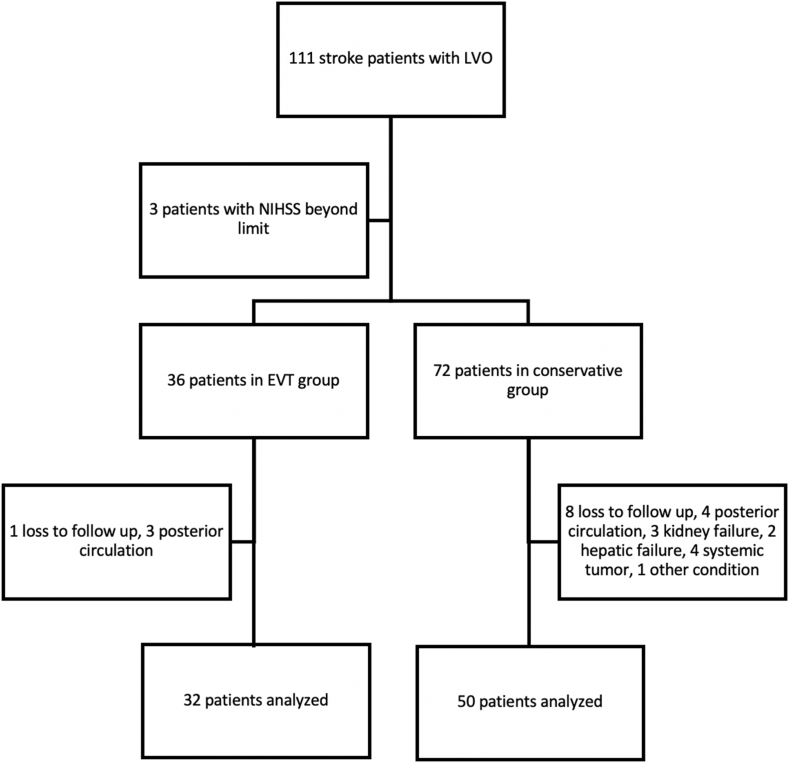
Subjects enrollment.

Between 2017 and 2021, we identified 111 patients with ischemic stroke due to LVO in our hospital. Three patients had their NIHSS below or over the designated range, hence excluded from our study. Our exclusion criteria were described in Fig. 1. Based on the inclusion criteria, there were only 32 patients in the EVT group and 50 patients in the conservative group included in this analysis.

Table 1 summarizes the baseline characteristics of 82 patients included in this study. As opposed to the conservative group, the patients in EVT group were significantly younger (p = 0.004) with lower proportion of hypertension (p < 0.001) and lower median systolic (p = 0.006) and diastolic (p = 0.016) values at admission. They also had earlier stroke onset than those in the conservative group (p = 0.023). Other classic vascular risk factors were similar between two groups. However, the patients in the EVT group had twice the chance of being intubated during their hospitalization period (RR 2.67; 95%CI 1.18–6.08; p = 0.014).

**Table 1:** Baseline characteristics (n = 82).

	EVT (n = 32)	Conservative (n = 50)	Overall (n = 82)	*P* value
Demographics				
Age (year)*	56.75 (12.21)	64.04 (10.15)	61.20 (11.50)	**0.004**^a^
Male sex^†^	23 (71.9)	28 (56.0)	51 (62.2)	0.148^c^
Medical history				
Hypertension^†^	16 (50.0)	43 (86.0)	59 (71.9)	**<0.001**^c^
Diabetes^†^	9 (28.1)	17 (34.0)	26 (31.7)	0.577^c^
Dyslipidemia^†^	6 (18.8)	17 (34.0)	23 (28.0)	0.134^c^
Atrial fibrillation^†^	6 (18.8)	6 (12.0)	12 (14.6)	0.399^c^
Smoking^†^	18 (56.3)	32 (64.0)	50 (61.0)	0.483^c^
**Clinical**				
Onset (mins)*	120 (30–1320)	180 (30–5760)	180 (30–5760)	**0.023**^b^
Door to CT scan (mins)*	26 (5–176)	27 (0–8640)	26 (0–8640)	0.196^b^
Blood pressure (mmHg)*				
Systolic	140 (107–170)	150 (100–270)	150 (100–270)	**0.006**^b^
Diastolic	86 (70–110)	90 (47–150)	90 (47–150)	**0.016**^b^
Intubation during hospitalization^†^	12 (37.5)	7 (14.0)	19 (23.2)	**0.014**^c^
NIHSS*	13 (8–24)	14 (6–22)	14 (6–24)	0.819^b^
TOAST Classification^†^				
LAA	2 (6.3)	3 (6.0)	5 (6.1)	0.123^d^
Cardioemboli	20 (62.5)	18 (36.0)	38 (46.3)	
Undetermined	10 (31.3)	29 (58.0)	39 (47.6)	
ASPECTS*	5 (0–10)	7 (0–10)	6 (0–10)	0.085^b^
Blood glucose level (mg/dL)*	131 (86–483)	142 (86–446)	137 (86–446)	0.384^b^
IVT^†^	14 (43.8)	21 (42.0)	35 (42.3)	0.876^c^

*Data are mean values with standard deviation in parenthesis or median values with minimum–maximum values in parenthesis.

^†^Data are actual values and proportion percentage in parenthesis.

a Student-t-test.

b Mann-Whitney-U test.

c Chi-Square test.

d Kolmogorov-Smirnov test.

Based on TOAST criteria, cardiac emboli was the most commonly identified stroke subtype (62.5 %) in the EVT group. After dichotomization, the proportion of cardiac emboli was statistically significant between two groups (OR 2.96; 95%CI 1.18–7.43; p = 0.019).

### 3.1 Primary outcome analysis

**Fig. 2:**
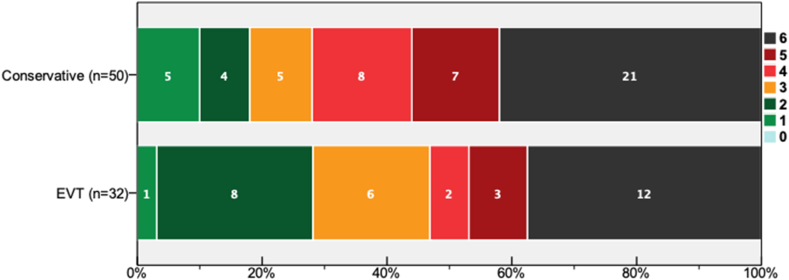
Functional outcome on day-90 from onset (n = 82).

Fig. 2 depicted the comparison of mRS at day-90. Based on *per-protocol* analysis, functional independence was observed in 28.1 % and 18.0 % in EVT and conservative groups, respectively. Despite its statistical insignificance (p = 0.280), patients within the EVT group showed higher trend of functional independence.

The number on the curve indicates actual number of patients in the designated group.

### 3.2 EVT subgroup analysis

An extended analysis on functional independence at day-90 in the EVT group was performed (Table 2). Both demographic characteristics and vascular risk factors between two groups were compared. The mRS ≤2 group presented with a lower mean NIHSS (p = 0.001). The mRS ≤2 group seemd to have earlier median onset from door-to-recanalization time although it was statistically insignificant.

**Table 2:** Functional independence predictors in endovascular therapy group (n = 32).

	mRS ≤2 (n = 9)	mRS >2 (n = 23)	*P* value
Age (years)*	52.89 (8.62)	58.26 (13.22)	0.191^a^
Male sex^†^	7 (77.8)	16 (69.6)	1.000
Medical History			
Hypertension^†^	5 (55.6)	11 (47.8)	1.000^d^
Diabetes^†^	3 (33.3)	6 (26.1)	0.685^d^
Dyslipidemia^†^	2 (22.2)	4 (17.4)	1.000^d^
Atrial Fibrillation^†^	2 (22.2)	4 (17.4)	1.000^d^
Smoking^†^	6 (66.7)	12 (52.2)	0.694^d^
Onset (mins)*	120 (30–1320)	180 (30–480)	0.409^b^
Onset to puncture (mins)*	360 (193–1731)	430 (177–810)	0.198^b^
Onset to recanalization (mins)*	435 (214–1861)	500 (272–980)	0.341^b^
Blood pressure (mmHg)*			
Systolic	140 (107–160)	140 (110–170)	0.281^b^
Diastolic	90 (70–110)	85 (70–100)	0.509^b^
Intubation during hospitalization^†^	1 (11.1)	11 (47.8)	0.103^d^
NIHSS*	12.22 (1.86)	14.78 (4.34)	**0.026**^a^
TOAST Classification^†^			
Cardioembolic	4 (44.4)	16 (69.6)	0.240^d^
Non-cardioembolic	5 (55.6)	7 (30.4)	
ASPECTS*	8 (2–10)	5 (0–10)	**0.001**^b^
Blood glucose level (mg/dL)*	140 (106–370)	130 (86–387)	0.433^b^
IVT^†^	4 (44.4)	11 (47.8)	1.000^d^
mTICI 2b/3^†^	7 (77.8)	13 (56.5)	0.422^d^

*Data are mean values with standard deviation in parenthesis or median values with minimum–maximum values in parenthesis.

†Data are actual values and proportion percentage in parenthesis.

^c^Chi-Square test.

^e^Kolmogorov-Smirnov test.

a Student-t-test.

b Mann-Whitney-U test.

d Fisher test.

Due to the limited number of patients, we only opted for NIHSS and ASPECTS to be further analyzed. In Table 3, ASPECTS was consistently a significant predictor of functional independence at day-90 in EVT group (p = 0.008).

**Table 3:** Multivariate analysis.

	Unadjusted OR (95%CI)	*P* value	Adjusted OR (95%CI)	*P* value
NIHSS at admission	0.81 (0.63–1.05)	0.112	0.87 (0.64–1.17)	0.349
ASPECTS at admission	2.43 (1.20–4.90)	0.013	2.43 (1.26–4.70)	0.008

## 4 Discussion

Most of our clinical decisions were made on clinical characteristics and plain brain CT scan. Our team implicitly chose the best candidate for our study by minimizing comorbidities. Firstly, the patients in the EVT group were younger. These results support the latest survey carried out by global neuro-interventionists where younger patients were more likely to receive EVT [11]. Younger patients also showed better functional recovery after 6 months [12]. Secondly, EVT group had lower incidence of hypertension. Despite the lack of association between hypertension and mortality in EVT, higher blood pressure at admission was associated with higher fatality and worse outcome [13]. On the other side, blood pressure should be lowered carefully to minimize secondary injury due to cerebral hypoperfusion [14]. Patients who required blood pressure control were more likely to be treated conservatively in our study.

The number of women who received EVT was 28.1 %. Our data did not imply that fewer women had intervention measures. Male sex had higher incidence of stroke both worldwide and in Indonesia [3,15]. The disparity in accessing EVT between male and female is beyond the scope of this study. Further research with larger data on all AIS patients is needed.

Due to its high cost, the patients in EVT group received ICU support after the procedure since they possessed a higher chance of requiring intubation when their general condition deteriorated. Powers et al. [16] found that 28 % of EVT patients required general sedation and intubation. Our study showed a similar finding. The indication of general sedation includes agitation, loss of consciousness, and respiratory failure due to sedative agents or the stroke itself [17]. General anesthesia was associated with lower mRS score due to collaterally damaging effects of hypotension and time delay [16,17]. Similar to the findings in our study, the proportion of intubation in the mRS >2 group was also higher.

Cardioembolic and undetermined strokes were the most identified subtypes in our study. The most common etiology in conservative group was undetermined stroke. Clinicians were often required to stabilize the patients; therefore, risk factors exploration was incomplete during hospitalization period even until death. Harris et al. [18] reported that the proportion of cardioembolism in our hospital was only 2.1 %. In their study, the data were not divided based on stroke severity. Further improvement in risk factor evaluation at our institution is warranted.

### 4.1 Functional independence at day-90 and its associated factor

Late presentation of stroke is a crucial problem that needs to be addressed. This issue was reflected in a very wide onset time interval. Unfortunately, a structured stroke referral was not available in our country. Continuous public awareness campaign and developing hospital network were some strategies we carried out to reduce pre-hospital delay [19]. Moreover, Asian culture relied on joint family discussion to decide clinical management which might contribute to time delay [20]. Other measures known to minimize such delay were deferred consent, onsite family member consent, and a more concise informed consent form [21].

Another problem to be solved was intra-hospital delay. It took at least 4 h from the patient's initial presentation at the emergency room to vascular catheterization. Patients with good functional outcome had earlier onset-to-puncture and onset-to-recanalization time by 1 h despite its statistical insignificance. Every hour of intra-hospital delay decreased 13 % chance of complete recanalization and 19 % chance of functional independence outcome [10]. The chance of complete recanalization and achieving functional independence after 8 h of stroke onset were similar to conservative therapy [22]. Thus, it resulted in harder thrombus due to its changing composition [23].

The ideal groin-to-recanalization time is 30 min [24]. The more proximal the occlusion site, the longer it takes for the operator to carry out the procedure. The proportion of good outcomes decreased by 70 % when the procedure time exceeds 60 min [24]. Jiang et al. [25] found onset-to-recanalization over 5 h to be a predictor of mortality. Therefore, such cumulative delay from emergency to catheterization room did impact recanalization success.

There were some factors that can help reduce intra-hospital delay, such as better coverage and higher volume when visiting patients, the presence of severe neurological deficits, patients’ arrival time (ideally within normal working hour), and longer onset time [26]. In the first quarter of IMS-III study, the median door-to-reperfusion time was 315 min which was similar to our study [21]. Some factors that reduced the likelihood of achieving time outlined in the clinical guideline were women, older age, and history of atrial fibrillation and diabetes mellitus [26]. Furthermore, Aghaebrahim et al. [27] introduced “no turn around” approach where patients were directly transferred to angiography room after imaging while withholding unnecessary procedures.

EVT effectiveness in large cohort studies had strict inclusion criteria where patients included in the study were those with the most likelihood benefit. Deb-Chatterji et al. [28] in Germany demonstrated that 26.2 % patients had functional independence after 90 days which was similar to our data. Although median age in EVT group was younger than HERMES [22], we also included patients with later onset of stroke, larger infarct core as reflected in lower ASPECT score, and longer time-to-puncture initiation. These patients would have been excluded from most big trials. ASPECT score was re-assessed retrospectively which could have contributed to non-significant functional independence. It was not unusual that a patient had a lower ASPECT score than once believed at admission. All patients treated were covered with Indonesian National Health Insurance. Limited cost coverage and lack of advanced imaging availability urged clinicians to make decisions based on no tissue perfusion data.

The prognostic factors that were consistently associated with functional outcome were age, time of onset, and NIHSS [29,30]. Initial NIHSS was associated with independent functional outcomes at day-90 [31,32]. Accurate NIHSS calculations in LVO has similar penumbra predictive capability to CT perfusion [33]. However, there is no standardized cut-off point for the NIHSS to predict good functional outcome [30].

The functionally independent group had a higher median ASPECT score. Each ASPECT score decrement resulted in 22.6 % lower odds of mRS score improvement after 90 days [28]. The rate of infarct volume growth relied on several factors, such as tissue tolerance, leptomeningeal collateral flow, physiological parameters, and comorbidities [34]. Penumbra evolution is a dynamic process between ischemic duration and collateral flow.

ASPECT scores were still assessed manually at our institution. Insula, internal capsule, and M2 cortex were areas known with low inter-rater concordance [35]. The use of narrow image windows helped improve reliability. Clinical-CT mismatch should be considered with simultaneous NIHSS and ASPECT score evaluation [36].

This study still owns several limitations. Firstly, this was a retrospective study; therefore, the workflow could not be controlled. ASPECT scores were retrospectively reassessed after the procedure. Secondly, sample size was small, hence matching could not be carried out. Patients included were also from a single institution, resulting in limited outcome generalization. Thirdly, other factors influencing post-stroke recovery, namely rehabilitation/neuro-restoration services and family support, were not considered. Nevertheless, EVT is a safe and helpful procedure that needs further reinforcement and support in Indonesia.

Hopefully, our findings provide insights and reassurance for health policy makers to improve hyperacute AIS care across the nation. Trainings and certifications need to be provided to more hospitals to allow greater patient access to comprehensive stroke hospitals. Furthermore, governments should give assurance to treating physicians that EVT costs are covered. In our situation, one major contributing intrahospital time delay was undue cost checking and coverage. We suggest to make “standard thrombectomy package” where unified thrombectomy and supporting tools are provided to all operators.

## 5 Conclusion

Our study showed a higher trend of functional independence at day-90 in the EVT group than conservative therapy in patients with AIS with LVO in CMH despite its statistical insignificance.

## Funding

This study was privately funded by all authors.

## Data availability

Supporting data and findings presented in this paper are available on request to corresponding author.

## Additional information

No additional information is available for this paper.

## CRediT authorship contribution statement

**Mohammad Kurniawan:** Writing – original draft, Supervision, Methodology, Investigation, Formal analysis, Data curation, Conceptualization. **Kevin Mulya Saputri:** Writing – review & editing, Writing – original draft, Visualization, Resources, Methodology, Investigation, Formal analysis, Data curation, Conceptualization. **Taufik Mesiano:** Supervision, Resources, Methodology, Investigation, Formal analysis, Data curation, Conceptualization. **Reyhan E. Yunus:** Investigation, Formal analysis, Data curation, Conceptualization. **Affan P. Permana:** Validation, Supervision, Resources, Investigation, Formal analysis. **Septo Sulistio:** Supervision, Methodology, Investigation, Conceptualization. **Eka Ginanjar:** Supervision, Formal analysis, Data curation. **Rakhmad Hidayat:** Supervision, Investigation, Data curation. **Al Rasyid:** Supervision, Formal analysis, Conceptualization. **Salim Harris:** Supervision, Methodology, Conceptualization.

## Declaration of competing interest

The authors declare that they have no known competing financial interests or personal relationships that could have appeared to influence the work reported in this paper.
